# Effects of Strain and Species on the Septo-Temporal Distribution of Adult Neurogenesis in Rodents

**DOI:** 10.3389/fnins.2017.00719

**Published:** 2017-12-19

**Authors:** Franziska Wiget, R. Maarten van Dijk, Estelle R. Louet, Lutz Slomianka, Irmgard Amrein

**Affiliations:** ^1^Division of Functional Neuroanatomy, Institute of Anatomy, University of Zurich, Zurich, Switzerland; ^2^Institute of Pharmacology, Toxicology and Pharmacy, Ludwig-Maximilian-University, Munich, Germany; ^3^Department of Health Sciences and Technology, ETH Zurich, Zurich, Switzerland

**Keywords:** doublecortin, Ki67, *Apodemus sylvaticus*, *Myodes glareolus*, *Mus domesticus*

## Abstract

The functional septo-temporal (dorso-ventral) differentiation of the hippocampus is accompanied by gradients of adult hippocampal neurogenesis (AHN) in laboratory rodents. An extensive septal AHN in laboratory mice suggests an emphasis on a relation of AHN to tasks that also depend on the septal hippocampus. Domestication experiments indicate that AHN dynamics along the longitudinal axis are subject to selective pressure, questioning if the septal emphasis of AHN in laboratory mice is a rule applying to rodents in general. In this study, we used C57BL/6 and DBA2/Crl mice, wild-derived F1 house mice and wild-captured wood mice and bank voles to look for evidence of strain and species specific septo-temporal differences in AHN. We confirmed the septal > temporal gradient in C57BL/6 mice, but in the wild species, AHN was low septally and high temporally. Emphasis on the temporal hippocampus was particularly strong for doublecortin positive (DCX+) young neurons and more pronounced in bank voles than in wood mice. The temporal shift was stronger in female wood mice than in males, while we did not see sex differences in bank voles. AHN was overall low in DBA and F1 house mice, but they exhibited the same inversed gradient as wood mice and bank voles. DCX+ young neurons were usually confined to the subgranular zone and deep granule cell layer. This pattern was seen in all animals in the septal and intermediate dentate gyrus. In bank voles and wood mice however, the majority of temporal DCX+ cells were radially dispersed throughout the granule cell layer. Some but not all of the septo-temporal differences were accompanied by changes in the DCX+/Ki67+ cell ratios, suggesting that new neuron numbers can be regulated by both proliferation or the time course of maturation and survival of young neurons. Some of the septo-temporal differences we observe have also been found in laboratory rodents after the experimental manipulation of the molecular mechanisms that control AHN. Adaptations of AHN under natural conditions may operate on these or similar mechanisms, adjusting neurogenesis to the requirements of hippocampal function.

## Introduction

There has been a long-standing interest in differences between the septal and temporal hippocampus, which are expressed by specific behavioral effects after septal or temporal lesions (e.g., Hughes, 1965; Moser et al., 1995), differences in the relative abundance of cell types (e.g., Gaarskjaer, 1978; Jinno and Kosaka, 2006), or efferent and afferent connections (Ishizuka et al., 1990; Agster and Burwell, 2013; Prasad and Chudasama, 2013). Such differences were typically thought to reflect gradual changes along an anatomical and functional septo-temporal continuum. Recently, detailed genomic studies (Thompson et al., 2008; Dong et al., 2009; Cembrowski et al., 2016a,b) suggest a functional differentiation within hippocampal principal cell populations (reviewed by Fanselow and Dong, 2010; Strange et al., 2014), which may subserve different functions in different septo-temporal segments. The septal hippocampus has been associated with cognition (Moser et al., 1995; Bannerman et al., 1999; Kjelstrup et al., 2008; Reichel et al., 2017). The intermediate hippocampus has been proposed to integrate the septally mediated visuospatial information with the motivational behavioral control for precise place learning which is translated into behavior (Bast, 2007; Bast et al., 2009; Barker et al., 2017). The interconnection of the temporal hippocampus with the amygdala (Felix-Ortiz et al., 2013) and its control of the HPA-axis (Herman et al., 1995; Lowry, 2002; Belujon and Grace, 2015) emphasizes its role in stress coping and anxiety-related behaviors (Fanselow and Dong, 2010).

In view of the functional differentiation of the hippocampal formation itself, it is not surprising that septo-temporal differences are also observed in one of the phenomena that it hosts—adult hippocampal neurogenesis (AHN). In most species that have been investigated, AHN is higher in the septal than in the temporal dentate gyrus (Uchida et al., 2005; Jinno, 2011b; Snyder et al., 2012; Amrein et al., 2015; Lowe et al., 2015). Interestingly, domestication selectively increases the number and distribution of doublecortin-positive neuroblasts and differentiating young neurons in the temporal dentate gyrus of foxes, a species in which temporal AHN is also higher than septal AHN (Huang et al., 2015). We have recently shown that AHN is a strong differentiator between rodent species (van Dijk et al., 2016a), in which the extent of AHN may relate to factors as diverse as habitat conditions (Cavegn et al., 2013) or social status (Oosthuizen and Amrein, 2016). The differentiation by AHN between species living under different natural conditions strongly implies an adaptive value. Considering the ontogenetic plasticity of AHN in response to experimental manipulation of the external or internal environment, it seems reasonable to expect that septo-temporal gradients in baseline AHN may be affected as well. Such gradients can become phylogenetically fixed if they are adaptive to the environment that represents a species niche. In this context, defining species-specific septo-temporal profiles for AHN in wild and laboratory-bred rodents may pinpoint, in which functional domain of the hippocampus one ought to look for the adaptive value of AHN.

Our data have, so far, been equivocal with regard to septo-temporal phylogenetic effects. While we did see such effects associated with domestication in foxes (Huang et al., 2015), we also found rather similar septo-temporal distributions of proliferating cells and differentiating young neurons in a comparison of C57BL/6 mice with New World monkeys (Amrein et al., 2015). In the present study, we therefore focused on two wild rodent species, which we previously found to differ from each other (Amrein et al., 2004a,b), that is bank voles (*Myodes glareolus*) and long-tailed wood mice (*Apodemus sylvaticus*). We also used F1 offspring of wild-captured house mice (*Mus domesticus*) to study if domestication may impact septo-temporal features of AHN in mice. Large differences in AHN were seen between different strains of laboratory mice (Kempermann et al., 1997; Hayes and Nowakowski, 2002; Kempermann and Gage, 2002b). We included C57BL/6 and DBA strains to compare them to the wild species and their wild-derived house mouse conspecifics. In DBA mice, far fewer new neurons are generated than in C57BL/6 mice (Kempermann et al., 2006; van Dijk et al., 2016b), and AHN reacts differently to experimental interference (Overall et al., 2013). Such strain differences may also extend to septo-temporal differences in AHN. For all mice, proliferation and neuronal differentiation were studied using Ki67 and doublecortin (DCX) as markers for proliferation and neuronal differentiation. To facilitate comparisons at different septo-temporal levels in different strains and species, these markers were applied to sections of extracted and physically extended hippocampi.

## Materials and methods

### Animals

Eighty four rodents of different strains and species were investigated (Table 1). C57BL/6, DBA2/Crl and house mice were 4.5–5.5 month old, while the age of wild trapped mice was unknown. All experimental work was conducted under the permits #26394 (laboratory and house mice) and #27034 (wild mice) of the Canton of Zürich veterinary office.

**Table 1:** Study animals.

**Species/strain**	**Number/sex**	**Source**	**Body weight (g)**	**Brain weight (g)**
C57BL/6	16 female	Charles River Laboratories, Germany	24.9 ± 2.0	0.48 ± 0.02
DBA2/Crl	16 female	Charles River Laboratories, Germany	24.3 ± 1.7	0.41 ± 0.01
House mouse *(Mus domesticus)*	13 female	Department of Evolutionary Biology and Environmental Studies, University of Zürich: F1 of wild trapped animals	24.5 ± 3.5	0.47 ± 0.04
Long-tailed wood mouse *(Apodemus sylvaticus)*	8 female 8 male	Wild trapped	*f* : 24.4 ± 3.9	*f* : 0.58 ± 0.03
			*m* : 28.3 ± 4.1	*m* : 0.59 ± 0.04
Bank vole *(Myodes glareolus)*	12 female 11 male	Wild trapped	*f* : 21.1 ± 3.4	*f* : 0.54 ± 0.06
			*m* : 24.3 ± 3.4	*m* : 0.52 ± 0.03

### Trapping and housing

Wild bank voles and wood mice were trapped at three locations (Zürich, Rifferswil, Trüllikon) in the canton of Zürich, Switzerland. Baited Sherman traps were set along hedges and scrubs from late afternoon to 4 h into darkness, traps were checked at 2–3 h intervals. Under isoflurane anesthesia, trapped animals were implanted subcutaneously with identification transponders (Planet ID GmbH, Germany) in the dorsal neck region, checked for health, gender, and signs of pregnancy, and treated against ectoparasites (Stronghold 15 mg, Selamectin, one drop). Animals were then single housed for 3 weeks. During this period, animals were fed *ad libitum* with anti-endoparasitic mouse food (Ivermectin, Kliba SA, Switzerland). Afterwards, animals were grouped according to species and sex. Groups of maximal nine adult animals were housed in a set of two to four tube-connected cages. All cages contained ample bedding and nesting material (tissue, hay, cardboard boxes) and *ad libitum* access to commercial mouse food and water. Group housed laboratory and house mice were kept in the same animal facility as wild mice. Lighting conditions corresponded to the natural light cycle in late summer (light on 07:00–19:00). All mice underwent the same behavioral testing in IntelliCages (Galsworthy et al., 2005) for 31 days before perfusion. Analysis of the behavioral tests is ongoing.

### Perfusion and dissection

Animals were deeply anesthetized with pentobarbital-Na intraperitoneally (50 mg/kg body weight) and perfused transcardially with 1% paraformaldehyde (PFA) solution containing 15% picric acid. Hippocampi were dissected, straightened and post-fixed in 4% PFA in this position for 5 h as described before (Amrein et al., 2015). The left and right hippocampi of each animal were randomly assigned to further processing.

### Gelatin embedding

For immunohistochemistry, one hippocampus of each animal was transferred into 20% glycerol in 0.1M phosphate-buffered saline (PBS, pH = 7.2) for cryoprotection overnight at 4°C. A gelatin matrix was used for tissue embedding as described in Smiley and Bleiwas (2012). Gelatin-egg albumin solution in 0.9% NaCl was prepared and mixed with 37% formaldehyde as hardener of the matrix. A 2.5M lysine solution and 25% glutaraldehyde at a ratio of 1:1 served as a cross-linking reagent. Mixed solution was poured as a base layer into embedding forms, four hippocampi were placed under the surface of the base layer. After setting of the base layer, a top layer of gelatin matrix was added. The hardened gelatin blocks were immersed in 20% glycerol. 40 μm sections were cut on a sliding microtom (HM 430, Thermo Fisher Scientific, Waltham, MA) equipped with a freezing stage. Sections were cut perpendicular to the hippocampal longitudinal (septo-temporal) axis and collected in 10 series. One series was collected in PBS in well plates and mounted in correct anatomical order (reference series). These sections were Giemsa stained (Iñiguez et al., 1985) (Giemsa stock solution 1.09204.1000. Merck, Darmstadt, Germany). Nine series were stored in cryoprotective solution (CPS) at −20°C until further processing.

### Immunohistochemistry

We used the endogenous proliferation marker Ki67 (Starborg et al., 1996; Cuylen et al., 2016; Sobecki et al., 2016) and the young neuron marker doublecortin (DCX; Matsuo et al., 1998). Test sections of all species and strains were used to titrate antibody concentrations that generated optimal signal-to-noise ratios and a saturated signal for the strongest stained cells. Every tenth section (one series) of each hippocampus was processed free-floating. Sections were rinsed in Tris-Triton (Tris-buffered saline (TBS), pH = 7.4 with 0.05% Triton). For epitope retrieval, sections were heat-treated for 45 min at 90°C in citrate buffer (Dako REAL, Glostrup, Denmark) diluted 1:10 in distilled water. Pre-incubation was done for 1 h in 2% normal horse serum (NHS) for Ki67 and 2% normal rabbit serum (NRS) for DCX in Tris-Triton with 0.2% Triton. Incubation with primary antibody in pre-incubation buffer followed over night at 4°C (Ki67: monoclonal mouse-anti-Ki67, BD Pharming, 1:1,000–1:3,000; DCX: polyclonal goat-anti-DCX, Santa Cruz, 1:4,000–1:8,000; for DBA mice in addition monoclonal mouse-anti-DCX, Santa Cruz, 1:50, and polyclonal rabbit-anti-DCX, Abcam, 1:1,000 were used). Thereafter, rinsed sections were incubated in biotinylated secondary antibody (all Vectastain Elite kits, Vector Laboratories, Burlingame, CA, USA, 1:1,000) diluted in TBS, 2% serum and 0.1% bovine serum albumin (BSA). Incubation in avidin-biotin-peroxidase complex (Vectastain Elite kits) was followed by staining with diaminobenzidine and H_2_O_2_ (Sigmafast™, D4418-50SET, Sigma-Aldrich, Steinheim, Germany) in distilled water. Mounted sections were embedded with Mowiol 4-88 (Ki67) diluted in glycerol and PBS (v/v) in a ratio of 1:3 (Sigma-Aldrich, Steinheim, Germany), or counterstained with hematoxylin solution (DCX) (Sigma-Aldrich, Steinheim, Germany) and embedded with Eukitt.

### Cell quantification

Quantification of the two markers Ki67 and DCX was performed applying design-based stereological methods (West et al., 1991, for stereology-specific parameters see Table 2). The investigations were carried out using the StereoInvestigator software v10.50 (MBF Bioscience, Williston, Vermont, USA) on a Zeiss Axio Imager 2 microscope, and blinded with regard to mouse identity, strain and species.

**Table 2:** Total estimated cell numbers (unilateral) and associated stereological parameters.

**Species/strain**	**Mean**	**Min**	**Max**	***SD***	**Mean CE = 0**	**CE^2^/CV^2^**	**Counting frame, μm (X, Y)**	**Sampling grid, μm(X, Y)**	**Disector height, μm**	**Evaluation interval**	**Mean sections analyzed**
**DCX**											
C57BL/6	5,954	4,087	9,750	1,250	0.09	0.19	45 × 45	75 × 75	40	10	14
DBA	1,418	660	3,070	638	0.07	0.02	Exhaustive counts		40	10	14
House mouse	3,192	1,028	8,111	1,708	0.13	0.06	45 × 45	75 × 75	40	10	13
Wood mouse	12,480	4,849	20,445	5,043	0.07	0.03	55 × 55	85 × 85	40	10	18
Bank vole	10,956	2,069	17,149	4,139	0.07	0.03	55 × 55	85 × 85	40	10	20
**Ki67**											
C57BL/6	3,811	2,550	6,180	820	0.06	0.08	Exhaustive counts		40	10	14
DBA	1,211	900	1,780	208	0.09	0.28			40	10	14
House mouse	1,752	780	2,780	599	0.08	0.06			40	10	13
Wood mouse	10,839	2,260	24,490	5,648	0.04	0.01			40	10	19
Bank vole	5,011	1,270	11,410	2,309	0.04	0.01			40	10	20

For the estimation of the total number of DCX-positive cells (DCX+), the optical fractionator of the StereoInvestigator software was used (West et al., 1991). Every tenth section was analyzed (section sampling fraction (ssf) = 1/10). Step sizes between sampling locations were determined using data from estimates of the area of the region of interest in pilot animals. Counting frame sizes (see Table 2) that returned, on average, more than one count from the sampling locations were determined by trials of differently sized frames. Cells were counted in the anatomically ordered sections using a 63× oil immersion objective (Zeiss, Plan-Apochromat/1.4 oil DIC). Nuclei of DCX+ cells were counted throughout the entire section thickness of 40 μm. To avoid oversampling, DCX+ cells that had their counterstained nucleus in focus in the focal plane that first touched the uppermost surface of the section were not counted. Following Disector counting rules (Sterio, 1984; West et al., 1991), only the nuclei of DCX+ cells that appeared in focus in the subsequent focal planes were included into the counts. We found DCX+ cells not only in the subgranular layer, but in some animals also within the granule cell layer. In eight randomly chosen animals, DCX+ cells in the subgranular layer were assessed separately from more superficial DCX+ cells between granule cells. Estimation of total cell number (N) for DCX+ cells was calculated using the following formula: $N=\;\sum Q^{-}\cdot \frac{1}{asf}\cdot \frac{1}{ssf}$, where ∑ *Q* ^−^ is the total number of counted cells. The area sampling fraction (asf) is calculated from the area of a counting frame, divided by the area associated with every step in x- and y direction ($\frac{a\left(counting\;frame\right)}{a\left(x,y\;step\right)}$).

Ki67-positive (Ki67+) proliferating cells in the subgranular layer of the dentate gyrus were counted exhaustively using a 63× oil immersion objective (Zeiss, Plan-Apochromat/1.4 oil DIC), again excluding Ki67+ nuclei that appeared in focus in the uppermost focal plane of a section. The anatomical order of the sections was determined by comparison of the sections with the reference series. Estimates of total Ki67+ cell number per hippocampus were calculated by multiplying the total counts by the section sampling fraction (ssf) of 10.

### Definition of the septal, intermediate and temporal part of the hippocampus

Acquisition of cell numbers were performed in the dissected and straightened hippocampi. Data of Ki67+ and DCX+ cells in each section in the correct anatomical order from septal to temporal were standardized for all animals into 12 virtual sections (bins) as described by Amrein et al. (2015). In short, raw data from each real section was divided into 12 sub-bins, corresponding to the desired number of virtual sections. Cell numbers for each virtual section was generated by successively pooling the cell numbers of these sub-bins. The number of pooled sub-bins corresponded to the number of the real sections available for an animal.

The division into equally sized septal, intermediate and temporal subdivision of the hippocampus was then applied on these 12 virtual sections. One third representing the septal (virtual sections 1–4), one third representing the intermediate (virtual sections 5–8) and one third representing the temporal (virtual sections 9–12) subdivisions of the hippocampus were used for graphical presentation and analysis.

### Statistical analysis

To assess estimate precision, the Coefficient of Error (*CE*) was calculated (Gundersen et al., 1999; Slomianka and West, 2005) for cell counts.

Statistical analyses and graphics were compiled using R software (version 3.3.2). The standardized data of Ki67+ and DCX+ cells within the septal, intermediate and temporal subdivision were used. To account for age-related differences between and within species and strains, values in the virtual sections were recalculated as the percentage of the total estimated cell number for each animal. Nested two-way ANOVA with gender as covariate was used to test for species/strain-specific differences in the relative distribution of Ki67+ and DCX+ cells in the three septo-temporal subdivisions. Tukey *post-hoc* analyses were performed when the main effect was found to be significant. Within species, the distribution of DCX+ cells and gender related differences was tested using a nested one-way ANOVA.

## Results

### Species-specific rates of AHN

Estimates of the total numbers of proliferating cells and young neurons were not statistically tested between species and strains, as the age of wild trapped animals was not assessed. The numerical data obtained (Table 2) do however confirm previously found species and strain differences. Wild house mice showed lower AHN than laboratory C57BL/6 (Klaus et al., 2012). Bank voles, lower in cell proliferation when compared to wood mice, had high numbers of DCX+ cells. This observation has been made previously in the comparison of bank voles with yellow-necked wood mice (Amrein et al., 2004b; van Dijk et al., 2016a), and may be due to attenuated cell death observed in this species (Amrein et al., 2004a). Proliferation in DBA laboratory mice was equal to previously reported data in this strain and at this age (van Dijk et al., 2016b), however DCX counts were unexpectedly lower. Repeating the estimates using three different DCX antibodies yielded similar results.

### Differences in septo-temporal AHN gradients

**Figure 1:**
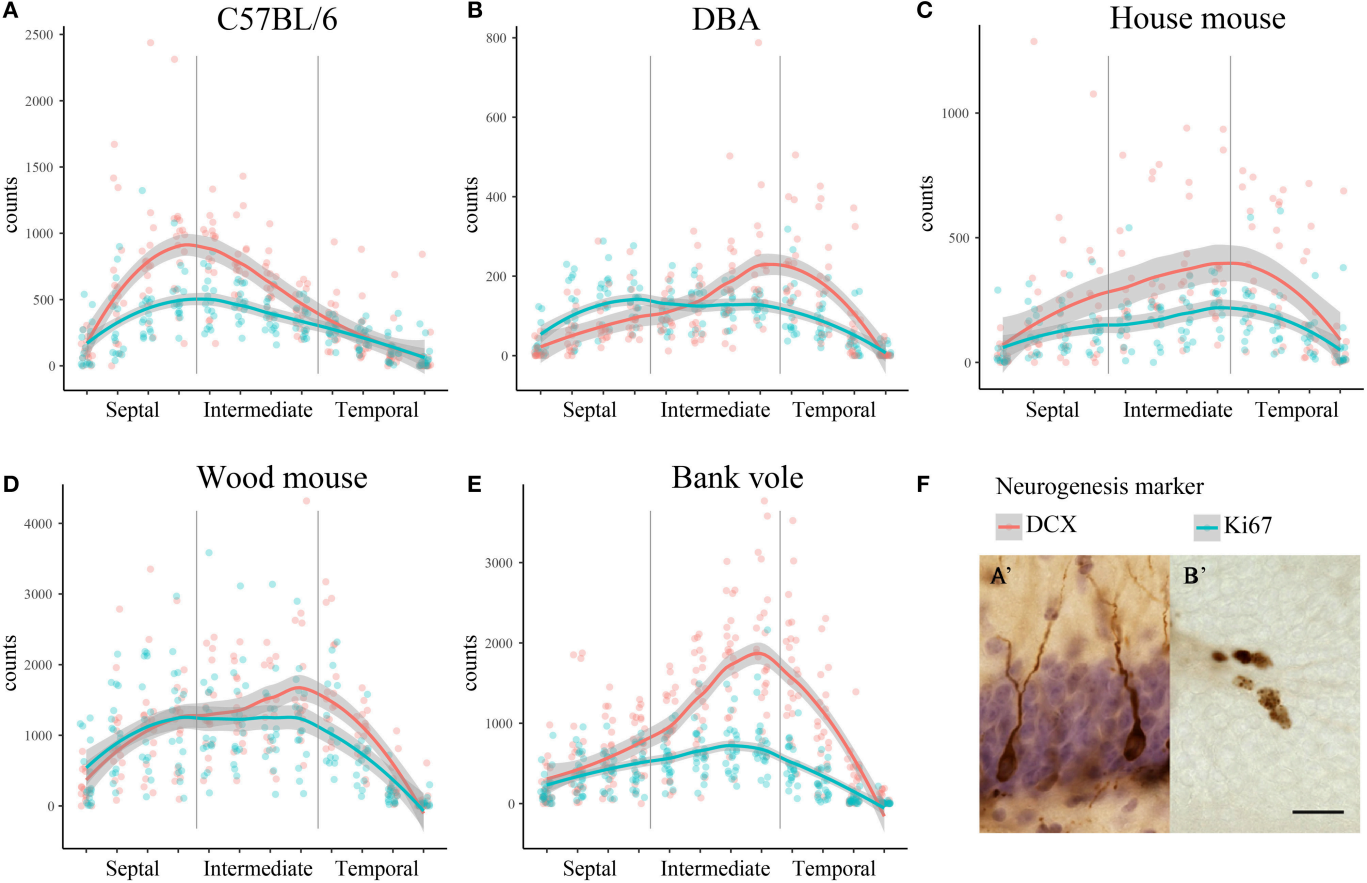
Distribution of neurogenesis-related absolute cell counts along the septo-temporal axis. Neurogenesis assessment in dissected, straightened hippocampi in laboratory C57BL/6 **(A)** follows a general septal>temporal gradient, whereas DBA **(B)**, house mice **(C)**, wood mice **(D)**, and bank voles **(E)** show a septal<temporal gradient for young neurons (DCX), and, except for DBA, also for cell proliferation (Ki67). Raw data of cell counts were re-distributed into 12 virtual sections to allow direct comparisons within and between species/strains, gray areas indicate 95% confidence intervals. **(F)** Shows representative images of hematoxylin-counterstained DCX+ young neurons **(A')** and Ki67+ cells **(B')** in the subgranular layer of the dentate gyrus. Scale bar = 20 μm.

**Figure 2:**
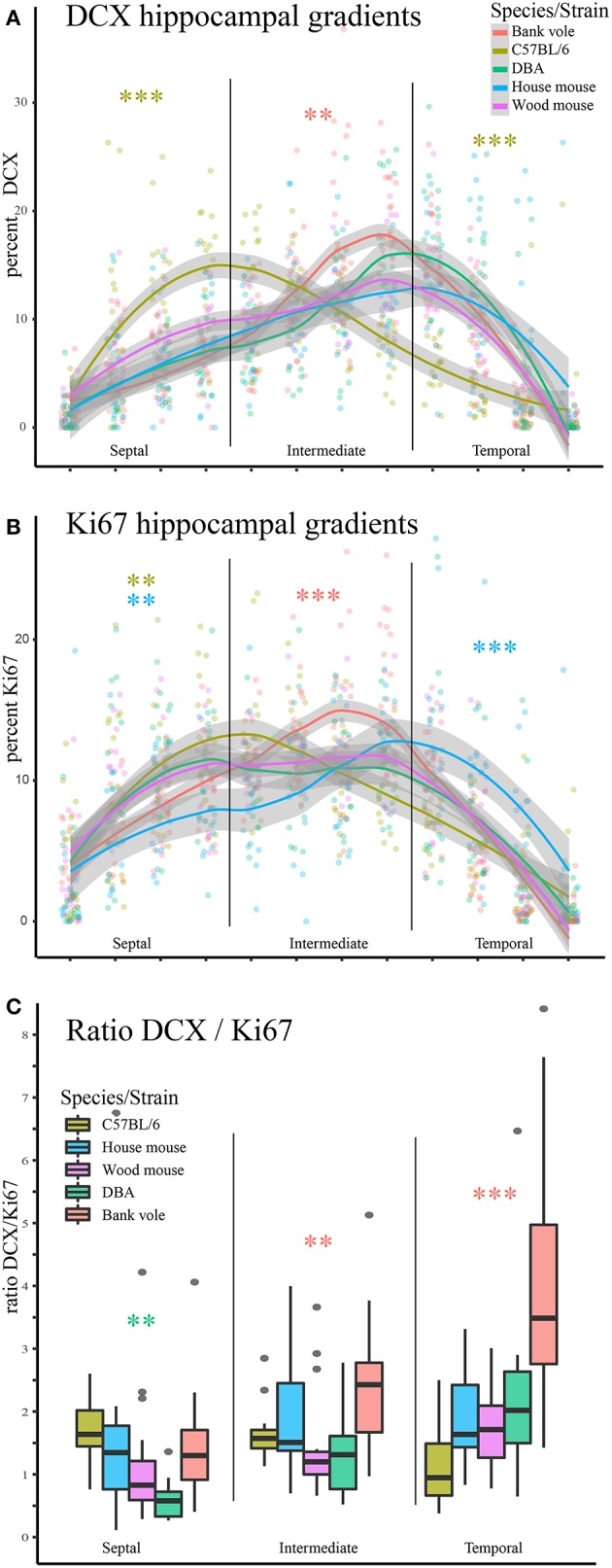
Opposite neurogenesis gradient in laboratory C57BL/6. Values of young neurons and proliferating cells were re-calculated as percentage for each animal to allow statistical comparisons. **(A)** C57BL/6 is distinct from other species and strains in the septal subdivision with significantly more young neurons than all other species (all *p* < 0.01) and significantly fewer young neurons in the temporal subdivision (all comparisons *p* < 0.05). In the intermediate hippocampus, bank voles have higher DCX values than C57BL/6, DBA and house mice. **(B)** Septo-temporal distribution of proliferating cells is overall more similar, however, in the septal subdivision the extremes are represented by C57BL/6 (high) and house mice (low), which are significantly different from DBA, bank voles and wood mice. In the intermediate hippocampus, bank voles score higher than all other species (*p* = 0.002 to < 0.0001). In the temporal subdivision house mice have more proliferating cells than all other species (*p* = 0.006 to < 0.0001). As a proxy for young neuron survival, the ratio of DCX+ cells to Ki67+ cells is presented in **(C)**, separately for the three hippocampal subdivision. Survival rate is lowest in DBA in the septal subdivision. Bank voles score higher in survival rate than DBA and wood mice in the intermediate (*p* < 0.01). In the temporal subdivision, neuronal survival in bank voles excels all other species values (*p* < 0.001). ^*^p < 0.05, ^**^p < 0.01, and ^***^p < 0.001; color-coding corresponds to the species/strain showing the difference, for exact values see results.

We observed striking differences in the distribution of proliferating Ki67+ cells and young neurons (DCX+ cells) along the septo-temporal axis in the five rodent species and strains (Figure 1). While C57BL/6 showed an emphasis on neurogenesis in the septal subdivision, all other rodent species had more neurogenesis toward the temporal hippocampus. Statistical comparisons on standardized data revealed a significant main effect for species and strains in the distribution of DCX+ cells (Figure 2A) along the hippocampal axis [*F*_(4, 77)_ = 11.36, *p* < 0.0001]. Furthermore, a strong interaction was found for species/strains and septo-temporal subdivisions [*F*_(8, 154)_ = 9.05, *p* < 0.0001]. *Post-hoc* comparison in the septal subdivision showed C57BL/6 to have higher DCX values than all other species (comparison wood mouse *p* = 0.004; bank vole, house mouse and DBA all < 0.0001), while DBA, bank voles, wood mice and house mice did not differ from each other. In the intermediate subdivision, bank voles showed higher DCX values than C57BL/6, DBA and house mice (*p* = 0.029, 0.01, and 0.004, respectively), the difference to wood mice did not reach significance (*p* = 0.07). All other pairwise comparisons were non-significant in the intermediate subdivision. C57BL/6 differ again in the temporal subdivision, by having significantly lower DCX values than all other species (wood mouse *p* = 0.049, bank vole 0.024, DBA and house mouse *p* < 0.0001). No further significant species differences were found in the temporal subdivision. We found no overall effect of species and strains in the distribution of Ki67+ cells (Figure 2B) along the septo-temporal axis [*F*_(4, 77)_ = 1.52, *p* = 0.21]. Interaction between species/strains and septo-temporal subdivision however was significant [*F*_(8, 154)_ = 7.46, *p* < 0.0001]. Within the septal subdivision, C57BL/6 showed higher Ki67 values compared to bank voles and house mice (*p* = 0.04 and *p* = 0.004). House mice on the other hand have significantly fewer Ki67+ cells than DBA and wood mice (*p* = 0.005 and 0.02). In the intermediate subdivision, bank voles had higher Ki67 values than all other species (*p* = 0.002 to < 0.0001). In the temporal subdivision, house mice differed from all other species in having the highest Ki67 values (*p* = 0.006 to < 0.0001). All animals had more DCX+ and Ki67+ cells in the intermediate part than in the septal or temporal subdivision (Table 3).

**Table 3:** Percentage of neurogenesis-related cells in the three hippocampal subdivision.

**Species/strain**	**DCX**			**Ki67**		
	**Septal (%)**	**Intermediate (%)**	**Temporal (%)**	**Septal (%)**	**Intermediate (%)**	**Temporal (%)**
C57BL/6	40	46	14	37	44	19
DBA	20	44	36	36	42	22
House mouse	19	43	38	24	42	34
Wood mouse	22	48	30	33	46	21
Bank vole	15	56	29	26	58	16

### Differences in the DCX/Ki67 ratio

As a proxy for young neuron survival, we also assessed the ratios of DCX+ cells to Ki67+ cells (Figure 2C). Overall, there are significant differences between strains and species [*F*_(4, 77)_ = 9.51, *p* < 0.0001] and an interaction between strain/species differences in the DCX/Ki67 ratio in the hippocampal subdivisions [*F*_(8, 154)_ = 10.97, *p* < 0.0001]. *Post-hoc* comparison for the septal subdivision suggests a lower survival rate in DBA compared to bank voles, house mice and C56BL/6 (*p* = 0.041, 0.028, and 0.006 respectively). In the intermediate subdivision, bank voles have a higher DCX/Ki67 ratio than wood mice and DBA (*p* = 0.009 and 0.003). Temporally, the DCX/Ki67 ratio of bank voles exceeded that of all other species (all *p* < 0.0001). All other comparisons did not show significant differences.

### Radial position of DCX+ cells in wild mice

**Figure 3:**
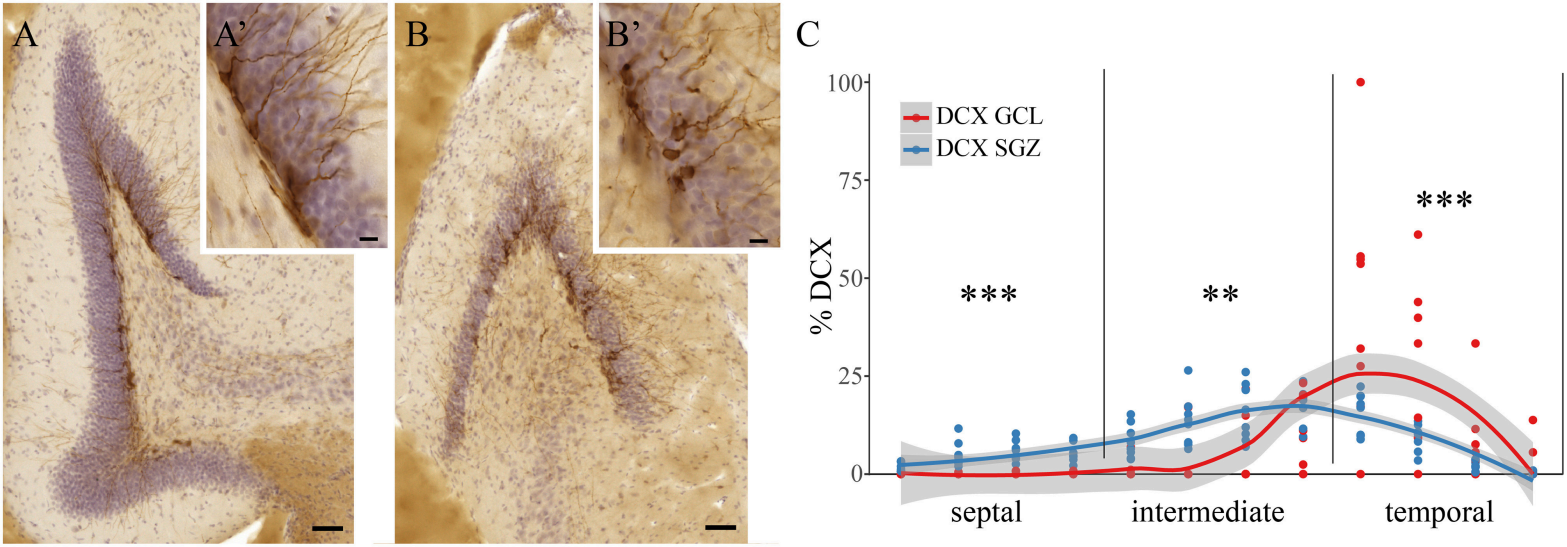
Radial location of DCX positive cells in bank voles and wood mice. DCX+ cells in wild rodents are not only found in the subgranular zone (SGZ, **A,A'**), but are found dispersed throughout the granular cell layer (GCL, **B,B'**). Quantitative assessment of DCX+ cells in the SGZ vs. those cells within the GCL reveals differences along the septo-temporal axis **(C)**. While DCX+ cells in the SGZ predominate in the septal and intermediate hippocampus, significantly more DCX+ cells are found within the granule cell layer in the temporal subdivision. Scale bare **(A,B)** = 50 μm; **(A',B')** = 10 μm. ^**^p < 0.01, and ^***^p < 0.001; for exact values see results.

Among all 84 quantified animals, some animals showed widely scattered DCX+ cells along the radial axis of the dentate gyrus. DCX+ cells were found in the subgranular zone (SGZ, Figures 3A,A') and in the dentate granule cell layer proper (GCL, Figures 3B,B'). Eight animals with this phenotype were randomly selected when animal identity was still blinded, and DCX+ cells were assessed separately in the SGZ and GCL. Out of the five investigated species, the selected animals exhibiting this phenotype were exclusively either wood mice or bank voles. The binomial probability of the sample to only contain wood mice and bank voles (*n* = 39) if this trait was distributed at random in all species and strains (*n* = 84) would be (39/84)^8^ = 0.002. Overall, we found in these two species a significant main effect of the radial axis distribution of DCX+ cells [*F*_(2, 14)_ = 17.8, *p* < 0.001] and a significant interaction between the radial distribution of young neurons and hippocampal subdivision (*p* < 0.0001). *Post-hoc* tests revealed that DCX+ cells in the SGZ predominate in the septal [*F*_(1, 14)_ = 50.7, *p* < 0.001] and intermediate [*F*_(1, 14)_ = 14.7, *p* = 0.002] subdivision, while in the temporal subdivision DCX+ cells found in the GCL proper are more common [*F*_(1, 14)_ = 36.2, *p* < 0.001, Figure 3C]. With the caveats of small samples and unequal group sizes, there was no difference between the two wood mice and six bank voles within this sample (data not shown).

### Sex-related differentiation along the septo-temporal axis in wood mice

**Figure 4:**
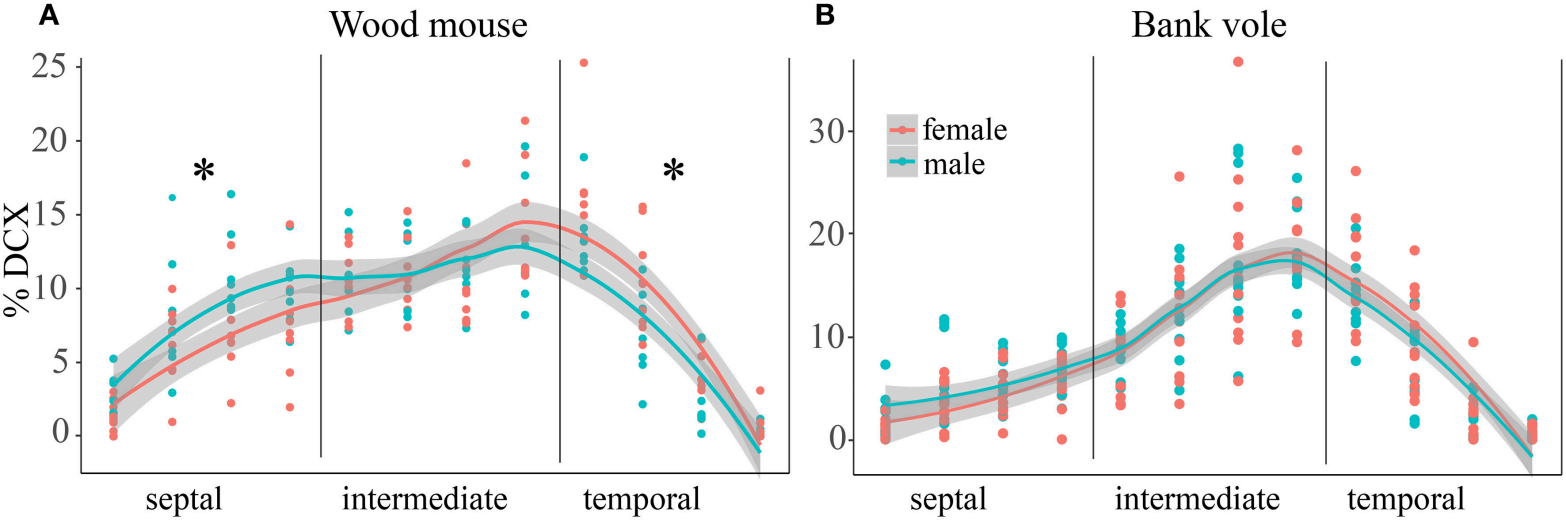
Sex differences in DCX+ cells in wood mice along the hippocampal axis. The septal < temporal gradient in DCX+ cell distribution is more pronounced in female wood mice **(A)** as compared to males. Septally, they have lower values than males, while temporally females DCX+ values exceed those of males. In bank voles **(B)**, males and females do not differ with regard to DCX+ cell distribution along the septo-temporal axis. ^*^p < 0.05; for exact values see results.

In wood mice and bank voles, animals of both sexes were available, and gender differences in distribution of proliferating and differentiating cells were tested for. The percentage of DCX+ young neurons did show a gender effect in wood mice for the distribution along the septo-temporal axis [*F*_(1, 14)_ = 9.91, *p* = 0.007, Figure 4A], and an interaction of gender with septo-temporal subdivisions [*F*_(2, 28)_ = 3.87, *p* = 0.033]. *Post-hoc* tests revealed that in the septal subdivision, female wood mice have lower DCX values then males (*p* = 0.03), the intermediate subdivisions did not differ (*p* = 0.62), and in the temporal subdivision females had higher DCX values (*p* = 0.0499). The percentage of Ki67+ cells did not show a gender effect [*F*_(1, 14)_ = 2.44, *p* = 0.14] in wood mice. We also found no gender × subdivision interaction for Ki67 [*F*_(2, 28)_ = 0.875, *p* = 0.43]. Likewise, bank vole female and males did not differ in the distribution of Ki67+ cells along the septo-temporal axis [*F*_(1, 21)_ = 0.046, *p* = 0.83], and there was also no gender x subdivision interaction [*F*_(2, 42)_ = 1.48, *p* = 0.24]. Gender differences for DCX in bank voles were absent as well [*F*_(1, 21)_ = 0.45, *p* = 0.51, Figure 4B]. Neither could interactions between gender and septo-temporal subdivisions be found in bank voles [*F*_(2, 42)_ = 1.42, *p* = 0.25].

## Discussion

### Opposing septo-temporal gradients of hippocampal neurogenesis in closely related rodents

Structural and functional specificity along the longitudinal axis of the hippocampus have been reported repeatedly (Fanselow and Dong, 2010; Kesner, 2013; Poppenk et al., 2013; Strange et al., 2014), with gradients of adult hippocampal neurogenesis (AHN) fitting well into this framework (Wu et al., 2015). The age related decline of AHN (Ben Abdallah et al., 2010; Amrein et al., 2011) has septo-temporally different effects on the relative densities of progenitor cell populations and a larger overall effect in the temporal dentate gyrus (Jinno, 2011a). The maturation of septally generated cells is faster than that of temporally generated ones (Piatti et al., 2011; Snyder et al., 2012), which, in turn, can be selectively accelerated by voluntary exercise (Piatti et al., 2011). Ritalin (Lagace et al., 2006), seizures (Ferland et al., 2002; Häussler et al., 2012) or GABA-receptor blockade (Felice et al., 2012) increase AHN more temporally than septally. Unpredictable stress results in a larger temporal decrease in AHN (Tanti et al., 2012), while acute stress appears to increase septal AHN (Kirby et al., 2013). Rats that display learned helplessness after inescapable shocks show a decrease of septal AHN, whereas AHN is not affected in rats that do not display this behavior (Ho and Wang, 2010). Behavioral effects specific to the ablation of AHN in the septal or temporal dentate gyrus have also been found (Wu and Hen, 2014). Different septo-temporal effects may be explained by the distribution of progenitors with septo-temporally differing stimulus sensitivities (Jhaveri et al., 2015) or suppressed activity of temporal stem cells (Sun et al., 2015).

Reports in rats, mice, gerbils and primates support a septal > temporal gradient for neurogenesis (Dawirs et al., 1998; Teuchert-Noodt et al., 2000; Snyder et al., 2009b, 2012; Jinno, 2011b; Amrein et al., 2015; Bekiari et al., 2015). Our data on C57BL/6 follow the same pattern. Neurogenesis is high septally and lowest in the temporal dentate gyrus. However, we observe the opposite gradient in DBA, house mice, wood mice and bank voles, in which neurogenesis is higher temporally than septally. The shift toward a higher temporal AHN is more pronounced in female wood mice as compared to male wood mice. The inverted gradient in these species is, to some extent, apparent in proliferation, neuronal differentiation and the ratio of DCX/Ki67 positive cells. As observed before in a larger and phylogenetically more diverse sample of species (Amrein et al., 2011), cell proliferation is less variable across species. It also appears more restricted with respect to a septo-temporal differentiation, as we found no main effect in the distribution of Ki67-positive cells between species. The largest differences between strains and species are observed for DCX+ young neurons. This cell population may either modulate local network circuitry or regulate hippocampus-dependent behaviors attributed to the septal or temporal hippocampus (Wu et al., 2015) according to species-specific needs. In particular, bank voles show a strong emphasis on a contribution of young neurons in the temporal hippocampal function. This is likely due to an increased or extended survival of young neurons in the temporal dentate gyrus.

### Neurogenesis and the hippocampal longitudinal axis

Gene expression studies (Thompson et al., 2008) do indicate a division into three domains, while behavioral (Moser and Moser, 1998) and electrophysiological studies (Patel et al., 2012, 2013) demarcate a temporal third from the (combined) septal-intermediate two-thirds (summarized in Strange et al., 2014). A smooth long-axis gradient as described for hippocampal connectivity (Amaral and Witter, 1989; Witter, 1993) and place field sizes (Kjelstrup et al., 2008) could have been a possible approach to analysis as well. Our partition of the hippocampus into equally sized subdivisions (septal, intermediate and temporal) is somewhat arbitrary, but roughly corresponds to the divisions that have been suggested and is independent of anatomical landmarks that may depend on the orientation of the sections when the hippocampus is cut *in situ*. Tanti and Belzung (2013) have previously emphasized the problems associated with comparisons of experimental outcomes that arise from variable *in situ* demarcations between septal and temporal hippocampus. Our approach to analyze dissected hippocampi cut perpendicular to their long axis allowed for comparisons between species with similar body sizes but different brain and hippocampal sizes with minimal data distortion. This approach has also been used previously to overcome the ambiguities of other definitions (Gaarskjaer, 1978; Rapp and Amaral, 1988; Jinno and Kosaka, 2006; Snyder et al., 2009b; Amrein et al., 2015; Sun et al., 2015). We took care to present the data in graphs visualizing the entire longitudinal axis. Beyond a septo-temporal differentiation, our data also indicate that neurogenesis shows few differences between strains or species in an intermediate subdivision and that this subdivision harbors the highest percentages of proliferating cells and young neurons in all species, accounting for ~50% of all Ki67+ and DCX+ cells. Research in gradients has focused primarily on the septal and/or temporal pole, either by investigating septal and temporal parts, or by dividing the hippocampus into two areas (Wu et al., 2015). To our knowledge, neurogenesis has not been experimentally manipulated selectively in the intermediate hippocampus. fMRI recordings of the optogenetically stimulated intermediate hippocampus (Weitz et al., 2015) indicate a special role of this hippocampal subdivision by its widespread yet distinct cortical and subcortical network activation. Specific inactivation of the intermediate hippocampus by lesions or pharmacogenetic deactivation in rats shows that the intermediate hippocampus is necessary for spatial components of episodic memory (Barker et al., 2017) and sufficient to translate rapid place learning into a behavioral response (Bast et al., 2009). Our finding of high neurogenesis in the intermediate hippocampus in all species and strains further emphasizes the importance of this subdivision in the context of AHN.

### Tame and wild

Defensive behavior, independent of prior experience, and adequate unconditioned fear responses in rats rely on an intact temporal hippocampus (Kjelstrup et al., 2002). There is a large body of evidence that a stress response is mediated by projections from the temporal subiculum to the basolateral amygdala and dorsomedial hypothalamus (reviewed by Lowry, 2002; Belujon and Grace, 2015). It should be pointed out here that the behavior of wild rodents brought into the laboratory is radically different from that of laboratory strains. Wild rodents do everything to avoid human contact and take painful defensive measures if it cannot be avoided. They are very skillful jumpers and will leap out of the cages for safety if given the smallest opportunity. Such behaviors are adaptive in the natural environment of wild mice, but selective pressures that result from the environment (e.g., predation) have been absent or even been replaced by selection for docility in the laboratory (Goto et al., 2013). The behavior of the F1 generation of wild house mice used here was indeed still very “wild-type” like. Differences in behavior should therefore be reflected in differences of temporal hippocampal function, and such differences apparently encompass a higher number of temporal generated young neurons in wild rodents used in this study. The unexpected wild-type-like temporal AHN in DBA mice may support this idea. Although poorer performers than C57BL/6 in many hippocampal-dependent spatial learning tasks (Ammassari-Teule et al., 1992; Jones et al., 2001), they show a better passive avoidance learning, which, in contrast to C57BL/6 mice, is not impaired by septal NMDA receptor antagonist infusion (Mineur et al., 2007; Baarendse et al., 2008). They also show less freezing in fear conditioning tasks than C57BL/6 mice (Baarendse et al., 2008) that is not altered by chronic mild stress (Mineur et al., 2007).

### DCX/Ki67 ratio, cell maturation and survival

Changes in the ratio between DCX+ and Ki67+ cell numbers may be generated by at least three different mechanisms. The duration of expressions of DCX or Ki67 in the cells may change. Ki67 is expressed during S, G2, and M phases of the cell cycle, but only late during G1. Considering the short duration of G1 in cycling progenitors (Lewis, 1978; Nowakowski et al., 1989; Overall et al., 2016), changes in Ki67 expression during G1 could not generate the DCX/Ki67 ratio differences seen in bank voles. Instead, there is evidence for differences between laboratory rats and mice in the rate of young neuron maturation (Snyder et al., 2009a). Furthermore, delayed maturation and extended expression of markers of young neurons has been described in other species (Amrein and Slomianka, 2010; Brus et al., 2013), including primates (Ngwenya et al., 2008, 2015; Kohler et al., 2011; Amrein et al., 2015). Differences in the speed of maturation of young neurons, depending on septo-temporal location, have also been reported in laboratory rodents (Piatti et al., 2011; Snyder et al., 2012). On this background, the high DCX/Ki67 ratio in the intermediate and, even more so, in the temporal dentate gyrus of bank voles suggest to us an extended period in which DCX is expressed in bank voles. In that a large fraction of newly generated neurons dies before expressing markers of adult neurons (Dayer et al., 2003; Kempermann et al., 2003), which coincides with the shut-down of DCX expression (Brown et al., 2003), the extended period of maturation may also represent an extended period of survival. An alternative explanation of differences in the DCX/Ki67 ratio would be differences between strains or species in the number of cells that will have either neuronal or glial fates (Kempermann and Gage, 2002a,b), although heritable traits have been found to account for only little of the differences in gliogenesis between strains (Kempermann et al., 2006). Our material does not allow the assessment of this possibility.

### Changes in the distribution along the radial axis

In wild wood mice and bank voles, our data suggest septo-temporal differences in the distance that DCX+ cells migrate along the radial axis, with DCX+ cells preferentially found in the SGZ in the septal and intermediate subdivision, and more DCX+ cells within the granule cell layer proper in the temporal hippocampus. In laboratory rodents, there is only a modest migration of newborn neurons into the dentate granule cell layer during the first week after their formation (Kempermann et al., 2003) and most of the newborn cells remain at the base or in the lower one-third of the granule cell layer. Both in sheep and monkeys, in which newly generated neurons retain immature characteristics far longer than in rodent, there is at least statistical evidence for a migration of new neurons into the granule cell layer (Kohler et al., 2011; Brus et al., 2013). Unfortunately, the illustrations provided do not allow a comparison with our observations in wood mice and bank voles. While a prolonged period of migration may result in differences in radial positioning, genetic control of cell migration can also generate a distribution of newborn cell reminiscent of that seen in the temporal dentate gyrus of wood mice and bank voles (Namba et al., 2011; Teixeira et al., 2012; Schafer et al., 2015; Woodbury et al., 2015). In the case of miR-155 overexpression, this seems possible even though the survival of newborn cells is reduced (Woodbury et al., 2015). We cannot address which of these factors may be responsible for the radial distribution of newborn cells in wood mice and bank voles. However, these mice show that natural selection may operate on these or similar mechanisms to generate what must be believed to be adaptive species differences.

## Concluding remarks

The variability in AHN in terms of absolute cell numbers, their septo-temporal distributions, ratios between proliferating and maturing cells between rodent species may be surprising and bewildering. It is less surprising when one considers the role that AHN may play in differentiating hippocampal function across taxonomic units. Within a larger sample of rodent species, we found that AHN is a far better differentiator between species than other hippocampal principal cell populations (van Dijk et al., 2016a). It is only equaled by variation in hilar cell numbers, which, like newborn neurons, are involved in the control of information flow through the dentate gyrus. Septo-temporal differences in AHN and differential septo-temporal effects of experimental manipulations suggest that AHN participates in the anatomical and functional septo-temporal differentiation of the hippocampus (Sahay and Hen, 2007; Glasper et al., 2012; Tanti and Belzung, 2013; Jinno, 2016). Considering the power of AHN to differentiate between species, a species-specific septo-temporal modulation of AHN turns from surprise to expectation. Much could be said about the relative vices and virtues of working with laboratory or wild rodents. Without the countless studies performed in laboratory animals, our observation would be phenomenology without plausible mechanisms. On the other hand, observations in wild animals may turn what we perceive as anatomical aberrations and functional deficits after the experimental manipulations of these mechanisms into substrates of adaptive onto- and phylogenetic changes.

## Author contributions

IA, RMvD and LS planned experiments. FW, EL, RMvD and IA conducted experiments FW, LS and IA wrote the manuscript.

### Conflict of interest statement

The authors declare that the research was conducted in the absence of any commercial or financial relationships that could be construed as a potential conflict of interest.
